# Monobutyrin Can Regulate the Gut Microbiota, Which Is Beneficial for the Development of Intestinal Barrier Function and Intestinal Health in Weaned Mice

**DOI:** 10.3390/nu16132052

**Published:** 2024-06-27

**Authors:** Haidong Wang, Ji Qiu, Minyao Zhou, Yanqiu Luo, Xinyu Li, Minqi Wang

**Affiliations:** The Key Laboratory of Molecular Animal Nutrition, Ministry of Education, College of Animal Sciences, Zhejiang University, Hangzhou 310058, China; wanghaidong@zju.edu.cn (H.W.); qiuji0121@zju.edu.cn (J.Q.); minyaoz@zju.edu.cn (M.Z.); 22117058@zju.edu.cn (Y.L.); lixinyu1502@zju.edu.cn (X.L.)

**Keywords:** monobutyrin, weaned mice, gut microbial, immune cell development, intestinal barrier function

## Abstract

In this study, we investigated the effect of monobutyrin (MB) on the gut microbiota and intestinal health of weaned mice. MB was administered via gavage to 21-day-old weaned mice. Samples of small intestinal and ileal contents were collected on day 1, day 7, and day 21 post-administration. Seven days of MB administration enhanced the mucin layer and morphological structure of the intestine and the integrity of the intestinal brush border. Both MB and sodium butyrate (SB) accelerated tight junction development. Compared to SB, MB modulated intestinal T cells in a distinct manner. MB increased the ratio of Treg cells in the small intestine upon the cessation of weaning. After 21 days of MB administration, enhancement of the villus structure of the ileum was observed. MB increased the proportion of Th17 cells in the ileum. MB facilitated the transition of the small intestinal microbiota toward an adult microbial community structure and enhanced the complexity of the microbial community structure. An increase in Th17 cells enhanced intestinal barrier function. The regulatory effect of MB on Th17 cells may occur through the intestinal microbiota. Therefore, MB can potentially be used to promote intestinal barrier function, especially for weaning animals, with promising application prospects.

## 1. Introduction

Epidemiological investigations have shown that newborns are more susceptible to infection than adults due to the immature or defective immune systems of newborns [1]. The intestinal mucosal immune system, as the largest immune organ, has gained extensive attention in recent years [2,3,4].

The weaning period, often referred to as the “window of opportunity”, is a critical phase in intestinal development. Goblet cell-associated antigen processing commences during the weaning period, allowing the immune system to encounter bacterial antigens [5]. The weaning period also represents a critical phase for the expansion of T-cell populations in the mouse small intestine [6,7]. The maturation of the microbiota and immune system can profoundly impact an individual’s health [8,9]. Microbiota disruptions impair the development of the intestinal immune system, increasing the susceptibility of the host to immune-related pathologies later in life [10,11,12]. Hence, maintaining the normal development of the gut microbiota and immune cells during the weaning period is particularly crucial for animal health.

Short-chain fatty acids (SCFAs) are metabolites produced by intestinal bacteria and have various immunomodulatory effects. They play a crucial role in the communication between immune cells and the intestinal microbiota [13]. Amongst the SCFAs, butyric acid can also ameliorate immune responses caused by infectious stimuli [14]. Butyric acid can also regulate the proportion of immune cells and enhance mucin and intestinal barrier function [15,16,17,18]. Monobutyrin (MB) is a modified butyric acid that overcomes several disadvantages of butyric acid, such as a pungent odor and susceptibility to spoilage.

In addition to being a butyric acid donor, MB, in its monomeric form, appears to have multiple functions. It can promote angiogenesis, inhibit *Clostridium difficile* proliferation, regulate the macrophage inflammatory response, and enhance epithelial barrier function [19,20,21,22]. Currently, research on MB in mice has been predominantly performed at the postweaning stage, with few studies focusing on the gut microbiota and intestinal health of mice during the weaning period. Mice are weaned at 21 days of age, and by 28 days of age, their gut microbiota structure approaches that of adults [23]. Thus, we studied newly weaned mice to explore the specific process by which intragastric MB administration impacts intestinal morphology development, tight junction functionality, microbial community maturation, and immune cell development. The aim of this study was to elucidate the mechanisms underlying the interaction between MB and the host microbiome, thereby laying the groundwork for future MB application in neonates or young animals.

## 2. Materials and Methods

### 2.1. Preparation of MB

MB was synthesized by J&K Scientific (90% purity, Beijing, China). Sodium butyrate (SB) was obtained from Shanghai Aladdin Biochemical Technology Co., Ltd. (98%, Cat# S102954, Shanghai, China). The MB and SB solutions used for intragastrical administration were prepared daily in phosphate-buffered saline (PBS, Cat# BL302A, Biosharp, Hefei, China). These solutions underwent ultrasonic emulsification for 30 min in an ultrasonic cleaner at room temperature. The dose of MB and SB was chosen according to our previous published study with some appropriate adjustment [24].

### 2.2. Animals and Experimental Protocol

This study was conducted according to the guidelines of the Institutional Animal Care and Use Committee of Zhejiang University (approval number: ZJU20230225; approval date: 27 June 2023).

A total of 96 healthy male 18-day-old C57BL/6 mice (11.96 ± 1.93) were obtained from the Zhejiang Academy of Medical Sciences (Hangzhou, China). All mice were housed in transparent plastic cages in a controlled environment with specific pathogen-free (SPF) conditions. The ambient conditions were 22 ± 3 °C, humidity controlled at 50 ± 10%, and a light and dark cycle of 12 h. All the mice had unrestricted access to standard ration (41% corn flour, 24% wheat flour, 10% fish meal, 8% bean cake, 8% bran, 4% alfalfa, 2% bone meal, 2% mineral, 1% vitamin) and water throughout the experiment. After three days of adaptation, the mice were randomly divided into PBS, MB, and SB groups based on their average body weight (BW). The mice in each group were intragastrically administered 0.4 mL of MB or SB at a dose of 1 g/kg BW (True dose). The intragastric administration was performed once daily at 10:00 A.M. Blood and tissue samples from the spleen, mesenteric lymph nodes, duodenum, jejunum, ileum, and cecum were collected from 24, 36, and 36 mice in each group on day 1 (8 mice in each group; 6 h after gavage), day 7 (12 mice in each group; 6 h after gavage), and day 21 (12 mice in each group; 6 h after gavage), respectively. The mice were anesthetized with isoflurane. Blood samples were collected from the retro-orbital plexuses. After blood samples were collected, the mice were euthanized with pentobarbital sodium. Intestinal sample identification was performed as described in the previous article [24]. Blood samples from the mice were placed in sterile centrifuge tubes at room temperature for 1.5 h to separate the serum and centrifuged at 4 °C and 3000× *g* for 10 min to obtain the upper serum layer. These serum samples were stored at −80 °C. For the isolation of lamina propria immune cells, intestinal samples were collected from half of the mice in each group. Intestinal samples (3–5 mm long) from the remaining half of the mice were gently rinsed with PBS and placed in appropriate tissue fixation solution for histopathological analysis. The remaining intestinal samples and ileal contents collected in a sterile centrifuge tube were stored at −80 °C for subsequent qPCR and 16S rDNA analysis.

### 2.3. Histological Staining

Duodenum, jejunum, and ileum segments of each group for histological staining were sampled at the same location. After being gently rinsed with PBS, segments of the duodenum, jejunum, and ileum were fixed in a 4% paraformaldehyde solution, followed by dehydration in a graded series of ethanol. After dehydration, the specimens were embedded in paraffin. Intestinal sections (6 μm thick) were stained with hematoxylin and eosin (H&E, Cat# AFIHC007, AiFang biological, Changsha, China). An optical microscope (Nikon Eclipse E100, Tokyo, Japan) and a Nikon DS-U3 (Tokyo, Japan) imaging system were utilized to observe intestinal morphology and acquire images. All villi and crypt in two consecutive complete transversal sections were measured. ImageJ software was used to measure villus height and crypt depth. The villus height-to-crypt depth ratio was calculated.

Alcian blue-periodic acid-Schiff (AB-PAS, Cat# C0142S, Beyotime Biotechnology, Shanghai, China) staining was performed to visualize goblet cells in the jejunum and ileum. Initially, the intestinal sections were deparaffinized, followed by staining with AB. After being rinsed with distilled water, the sections were subjected to oxidation in a periodic acid solution and subsequently stained with Schiff reagent. Goblet cells in all villi from two consecutive complete transversal sections were counted. The observation of the stained sections and the image capture equipment used were the same as described for the H&E-stained sections.

### 2.4. Transmission Electron Microscopy (TEM)

Ileal segments were rinsed with PBS and subsequently fixed in a 2.5% glutaraldehyde solution (Cat# 30092436, Sinopharm Chemical Reagent, Shanghai, China) at 4 °C for 24 h. Afterward, all the samples were washed three times in PBS for 15 min each. Postfixation was performed using a 1% osmic acid solution (Cat# 20816-12-0, SPI-Chem, West Chester, PA, USA) for 2 h. The samples were dehydrated through incubation in gradient concentrations of ethanol (30%, 50%, 70%, 80%, 90%, and 95%) for 15 min at each step, with the final step of dehydration in 100% ethanol (Cat# 10009218, Sinopharm Chemical Reagent, Shanghai, China) for 20 min. Subsequently, the samples were treated with 100% acetone (Cat# 10000418, Sinopharm Chemical Reagent, Shanghai, China) before being embedded in a pure Spurr’s resin mixture (Item# 02680-AB, SPI-Chem, PA, USA).

The embedded samples were sliced into sections with a thickness of 70–90 nm using an ultramicrotome (Leica Em Uc7, Leica, Wetzlar, Germany). These sections were counterstained with uranyl acetate (Item# 02624-AB, SPI-Chem, PA, USA) and lead citrate (Cat# L885990, Macklin, Shanghai, China). Finally, images of the sections were captured and observed using a Hitachi HT7650 electron microscope (Hitachi, Tokyo, Japan).

### 2.5. Quantitative RT‒qPCR (qPCR)

Approximately 100 mg intestinal tissue was used for quantitative total RNA extraction. The RNA extraction and quantitative RT‒PCR (qPCR) was conducted following the procedures described in our previous study [24]. The calculation of mRNA expression levels was performed using the 2^−ΔΔt^ method. GAPDH was selected as the housekeeping gene. Information about the primers used is provided in Table S1.

### 2.6. Jejunum and Ileum Lamina Propria Cell Isolation

The isolation of lamina propria cells from the jejunum and ileum was conducted following the protocol described by Scott et al., with some modifications [25]. The jejunum and ileum were dissected longitudinally and washed twice in PBS solution containing 2% fetal bovine serum (FBS, Cat# 40130ES76, Yeasen Biotechnology, Shanghai, China), 10 mM of HEPES (Cat# 15630080, Thermo, Waltham, MA, USA), and 2 mM of EDTA (Cat# AM9260G, Thermo, MA, USA). The washed jejunum and ileum sections were segmented into lengths of 3–5 cm and shaken in the aforementioned PBS solution at 37 °C and 250 rpm for 20 min. The shaking procedure was repeated twice. Then, the intestinal pieces were further washed twice with PBS solution containing 2% FBS. The jejunum and ileum sections were then minced into smaller fragments using sterile surgical scissors. Subsequently, the cells were digested in RPMI 1640 medium (Cat# SH30096.01, Hyclone, Logan, UT, USA; containing 10% FBS, 2 mM of L-glutamine (Cat# 25030081, Thermo, MA, USA), 100 U/mL of penicillin, 100 μg/mL of streptomycin (Cat# C3420-0100, Viva cell, Shanghai, China), 0.6 mg/mL of collagenase VIII (Cat# 9001-12-1, Sigma-Aldrich, St Louis, MO, USA), and 150 μg/mL of deoxyribonuclease I (Cat# D8071, Solarbio Science & Technology, Beijing, China)) at 37 °C for 60 min. The digested small intestine tissues were then passed through 100 μm and 40 μm cell strainers. Following centrifugation at 400× *g* for 6 min, lamina propria cells from the intestinal tissue were obtained.

### 2.7. Flow Cytometry

The isolated intestinal lamina propria cells were stained with Fixable Viability Dye 510 (FVS510, Cat# 564996, B&D, San Jose, CA, USA) for 15 min at room temperature and washed twice in PBS containing 2% FBS. The cells were then stained with the fluorescence-labeled surface markers CD45-APC-Cy7 (Cat# 557659, B&D, San Jose, CA, USA), TCR-β-FITC (Cat# 553170, B&D, San Jose, CA, USA), CD4-PerCP/Cyanine5.5 (Cat# 116012, BioLegend, San Diego, CA, USA), and EPCAM-APC (Cat# 17-5791-82, Thermo, Waltham, MA, USA). For intracellular transcription factor staining, the cells were fixed and permeabilized for 1 h using a transcription factor buffer (Cat# 562574, B&D, San Jose, CA, USA). After permeabilization, the cells were stained for 1 h with the following intracellular markers: Foxp3-PE-Cy7 (Cat# 25-5773-82, Thermo, Waltham, MA, USA), T-bet-BV421 (Cat# 563318, B&D, San Jose, CA, USA), and RORγt-PE (Cat# 562607, B&D, San Jose, CA, USA). Flow cytometry analysis was performed using a FACS Verse (B&D Systems, San Jose, CA, USA). Cells were first gated on FSC-A versus SSC-A, and single cells (SSC-A versus SSC-H), live cells (FSC-A versus FVS510^−^), and CD45^+^EPCAM^−^ lymphocytes were gated. T cells (TCRβ^+^CD4^+^) were gated from CD45^+^EPCAM^−^ cells. Treg cells (SSC-A versus Foxp3^+^), Th1 cells (SSC-A versus T-bet^+^), and Th17 cells (SSC-A versus RORγt^+^) were gated from CD45^+^EPCAM^−^TCRβ^+^CD4^+^ cells. This flow cytometry analysis was performed to characterize specific immune cell populations among lamina propria cells isolated from the intestine. The gating strategy for flow cytometry is shown in Figure S3.

### 2.8. Ileal Contents DNA Extraction and 16S rDNA Sequencing

Microbial DNA was obtained from the ileal contents using the cetyltrimethylammonium bromide (CTAB) method. The concentration and purity of the DNA were assessed using 1% agarose gels. Subsequently, the DNA concentration was adjusted to 1 μg/μL with sterile water. The amplification of the V3–V4 gene region of the bacterial 16S rDNA gene was carried out using the 341F (5′-CCTAYGGGRBGCASCAG-3′) and 806R (5′-GGACTACNNGGGTATCTAAT-3′) primer set. Polymerase chain reaction (PCR) amplification was performed using a Phusion^®^ High-Fidelity PCR Master Mix Kit (Cat# E0553S, New England Biolabs, Ipswich, MA, USA). Following electrophoresis on a 2% agarose gel, the PCR products were purified using a Qiagen Gel Extraction Kit (Cat# 28704, Qiagen, Hilden, Germany). Sequencing libraries were generated using a TruSeq^®^ DNA PCR-Free Sample Preparation Kit (Cat# 20015963, Illumina, San Diego, CA, USA) and qualified with a Qubit@ 2.0 Fluorometer (Thermo, Waltham, MA, USA) and an Agilent Bioanalyzer 2100 system. The 250 bp paired-end reads were obtained through sequencing on the Illumina MiSeq platform at Novogene Technology Co., Ltd. (Beijing, China). The paired-end reads were trimmed for the barcode and primer sequences and subsequently merged using FLASH (version 1.2.11) [26]. Following filtration and the removal of chimeric sequences, clean reads were obtained and used for subsequent analysis.

### 2.9. Bioinformatics Analysis of 16S rDNA Sequencing Data

The reads were processed in QIIME2 (version 2023.2) for denoising, phylogenetic tree construction, and taxonomic classification [27]. Specifically, DADA2 was used to denoise the sequences and generate amplicon sequence variants (ASVs) [28]. A phylogenetic tree was constructed using FastTree [29]. Taxonomic classification was performed using the full-length Greengenes2 (version 2022.10) database to annotate a total of 56,071 sequences [30]. The α-diversity and β-diversity (based on principal coordinate analysis (PCoA) results using weighted/unweighted UniFrac distances) of the microbial communities on the basis of the 16S rDNA sequencing results were analyzed using the web-based tool MicrobiomeAnalyst (www.microbiomeanalyst.ca; accessed on 8 October 2023) [31]. Illustration of the relative abundance of microbial taxa was performed by Origin software (version 2022, OriginLab Corporation, Northampton, MA, USA). Linear discriminant analysis effect size (LEfSe) analysis was conducted within the Docker image of LEfSe (https://github.com/biobakery/biobakery/wiki/lefse; accessed on 16 October 2023) [32]. The LDA score and *p* value thresholds were set to 2 and 0.1, respectively. The microbial age of the ileal microbiota was calculated according to the method of Gao et al. [33]. In brief, the R package Random Forest (version 4.7-1.1) was utilized to determine whether the gut microbiota correlated with age in the PBS group. Subsequently, a list of microbial taxa ranked by “feature importance” was generated in the report. Following this, the “rfcv” function from the R package Random Forest was applied for 100 iterations of cross-validation to determine the minimum number of microbiota constituents required for age prediction. This model was then applied to predict microbial age in other treatment groups, with the results presented on a graph with actual age on the *X*-axis and predicted microbial age on the *Y*-axis. In a co-occurrence network analysis, the taxa whose relative abundance was greater than 0.01% of the total taxa and which were present in more than 50% of the samples were first selected at the genus level. Subsequently, the R package WGCNA (version 1.72-1) was utilized for correlation analysis to identify taxa with a correlation coefficient |r-value| > 0.6 and a *p* value < 0.01. These selected taxa were used to construct the co-occurrence network. For co-occurrence network construction and the calculation of topological parameters, the R package igraph (version 1.5.1) was used. The topological parameters encompass various indices, such as proportion of positive correlation, proportion of negative correlation, average degree, average path length, and clustering coefficient. The network was visualized using Gephi software (version 0.9.2), and the Fruchterman–Reingold layout was applied for visualization. Spearman correlation analysis was conducted using the R package “psych” (version 2.3.9).

### 2.10. Relative Quantification of Segmented Filamentous Bacteria (SFBs) in the Ileal Mucosa

SFB genomic DNA was extracted from the ileal mucosa using a MonPure™ Universal Genome DNA Kit (Cat# MI00101S, Monad Biotech Co., Ltd., Suzhou, China). The concentration of the ileal mucosa DNA was determined by a NanoDrop 2000 spectrophotometer (Thermo Fisher Scientific, Waltham, MA, USA). Primers were designed for the 16S rDNA of SFB, and fragments amplified with Eubacteria (EUB) primers were used as internal reference genes. qPCR was conducted as previously described. The relative abundance of SFB was calculated by the 2^−ΔΔCt^ method. Information about the primers used is provided in Table S1.

### 2.11. Statistical Analysis

All the data are presented as the means ± standard errors of the means (SEMs). Statistical analyses were conducted using GraphPad Prism 8.0 (Boston, MA, USA) or SPSS 20.0 (Chicago, IL, USA). The normality of the data was tested using the Shapiro–Wilk test. Depending on the distribution of the data, one-way analysis of variance (ANOVA) or the Kruskal‒Wallis test (nonparametric) was used for analysis, followed by Tukey’s post hoc comparison (* *p* < 0.05; ** *p* < 0.01). Flow cytometric data were analyzed with FlowJo software version 10.8.1 (B&D, San Jose, CA, USA).

## 3. Results

### 3.1. Effect of MB Administration on the Intestinal Morphological Structure

H&E staining was conducted to investigate the effects of MB and SB on intestinal morphological structure (Figure S4). On the first day of the intragastric administration experiment, MB and SB did not have a significant effect on the morphological structure indicators of the duodenum or jejunum (*p* > 0.05). However, the villus height of the ileum was significantly reduced by intragastric SB administration (*p* < 0.05). On day 7, SB administration significantly decreased the duodenal crypt depth (*p* < 0.05). MB increased the duodenal villus height/crypt depth ratio (*p* < 0.05). In addition, MB decreased the jejunum crypt depth (*p* < 0.05). The intestinal morphological structure indicators of the duodenum and jejunum did not change significantly after 21 days of treatment with MB or SB (*p* > 0.05). However, MB significantly increased the ileal villus height (*p* < 0.05). In conclusion, SB may stimulate the intestine of newly weaned mice after 1 day of intragastric administration. MB administration for 21 days mainly affected the ileum of the mice.

### 3.2. Effect of MB Administration on the Small Intestinal Mucus Layer

**Figure 1:**
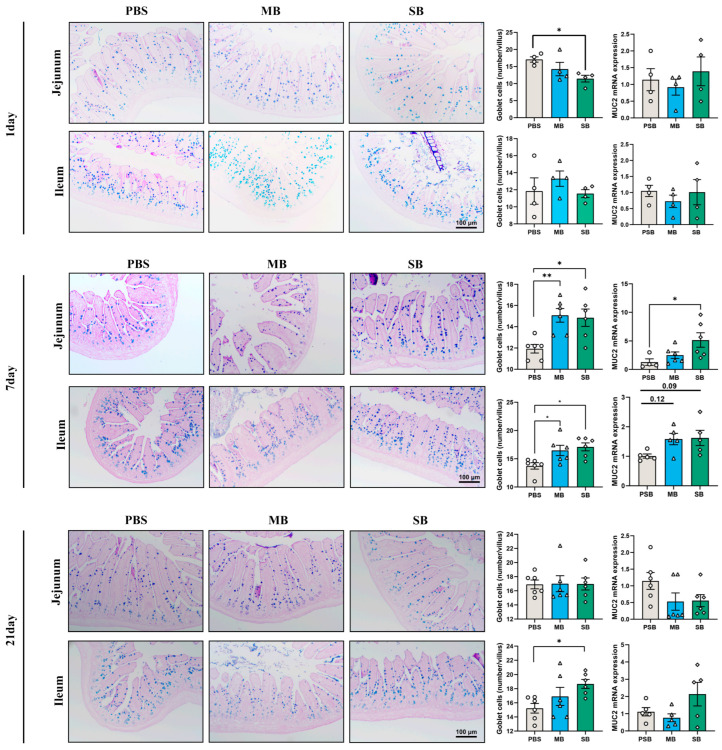
The number of goblet cells and MUC2 mRNA expression in the small intestines of mice on 1, 7, and 21 days after MB and SB administration. The left side shows the AB-PAS staining observation of the intestines under different intragastric administration days (1 day, n = 4; 7 days, n = 6; 21 days n = 6), and the right side shows the number of goblet cells and MUC2 mRNA expression. Scale: 100 μm. MUC2 mRNA expression (1 day n = 3; 7 and 21 days, n = 5). * and ** indicate significant difference (*p* < 0.05 and *p* < 0.01, respectively).

MUC2, which is secreted by goblet cells, is one of the major components of the mucus layer [34]. As shown in Figure 1, at one day after the intragastric administration of MB or SB, MB had no significant effect on the number of goblet cells in the jejunum or ileum (*p* > 0.05). In contrast, SB significantly reduced the number of goblet cells in the jejunum (*p* < 0.05). In addition, MB and SB had no significant effect on MUC2 mRNA expression in the jejunum or ileum (*p* > 0.05). On day 7, both MB and SB administration significantly increased the number of goblet cells in the jejunum and ileum (*p* < 0.05). SB significantly increased MUC2 mRNA expression in the jejunum of mice (*p* < 0.05). MB had no significant effect on MUC2 mRNA expression in the jejunum or ileum (*p* > 0.05). After 21 days of intragastric administration, only SB increased the number of ileum goblet cells (*p* < 0.05), whereas MB administration had no significant effect on the number of goblet cells or the expression of MUC2 mRNA in the jejunum or ileum (*p* > 0.05). These results suggest that SB can stimulate the mucus layer of the small intestine after 1 day of intragastric administration, which is consistent with the intestinal morphology results (Figure S4). MB increased the number of goblet cells after 7 days of gavage, while only SB administration altered the number of ileum goblet cells after 21 days of administration.

### 3.3. Effect of MB Administration on the Tight Junction and Brush Border

**Figure 2:**
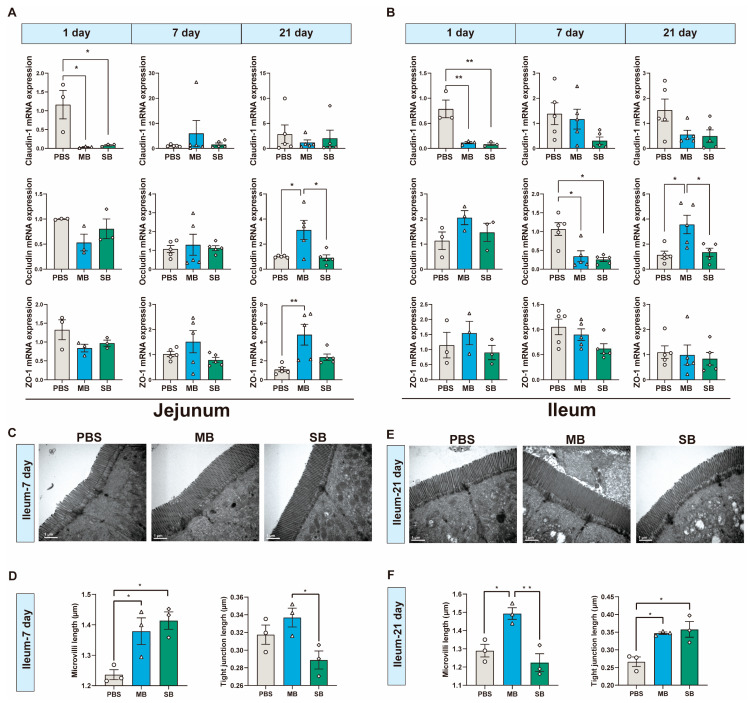
Effect of MB and SB on the tight junction and brush border of the small intestines on 1, 7, and 21 days after administration. (**A**) Jejunum tight junction protein mRNA expression. (**B**) Ileal tight junction protein mRNA expression (1 day, n = 3; 7 and 21 days, n = 5). (**C**,**D**) Effect of MB and SB on the brush border of ileal epithelial cells after 7 days of administration. (**E**,**F**) Effect of MB and SB on the brush border of ileal epithelial cells after 21 days of administration. Three intestinal samples were selected for each treatment, 3−4 fields of view were selected from TEM images of each mouse for statistical analysis, and 10 microvilli and 1 tight junction structure were randomly selected for each photo for length measurement. Microvilli length (n = 3); tight junction length (n = 3). * and ** indicate significant difference (*p* < 0.05 and *p* < 0.01, respectively).

We assessed the mRNA expression of genes associated with tight junctions. In addition, for the ileum, we used TEM to observe the microstructure, including brush borders and tight junction structures (Figure 2). After 1 day of intragastric administration, both MB and SB significantly decreased the expression of claudin-1 mRNA in the jejunum and ileum of the newly weaned mice (Figure 2A,B, *p* < 0.01). However, there was no significant effect on the mRNA levels of occludin or ZO-1 (Figure 2A,B, *p* > 0.05). After 7 days of intragastric administration, they had no significant effect on jejunal occludin mRNA expression (*p* > 0.05) but did significantly decrease ileal occludin mRNA expression (Figure 2A,B, *p* < 0.05). After 21 days of MB and SB administration, MB administration significantly increased jejunal ZO-1 mRNA expression and jejunal and ileal occludin mRNA expression (*p* < 0.05). Therefore, we selected the ileum at 7 and 21 days after intragastric administration for TEM observation and analysis (Figure 2C–F). The results showed that at both time points, the brush border structure remained intact, well organized, and densely arranged. The tight junction structures were intact without obvious structural damage (Figure 2C,E). After 7 days of intragastric administration, both MB and SB significantly increased the ileum microvilli length (Figure 2D, *p* < 0.05). After 21 days of treatment, MB significantly increased the ileum microvilli length, and both MB and SB significantly increased the tight junction length of the ileum.

### 3.4. Effect of MB Administration on Cellular Immune Development

#### 3.4.1. Effect of MB and SB Administration on the Proportion of Immune Cells

**Figure 3:**
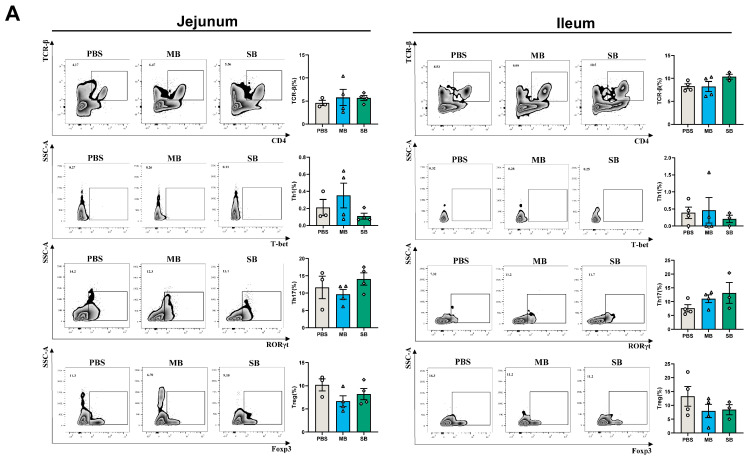

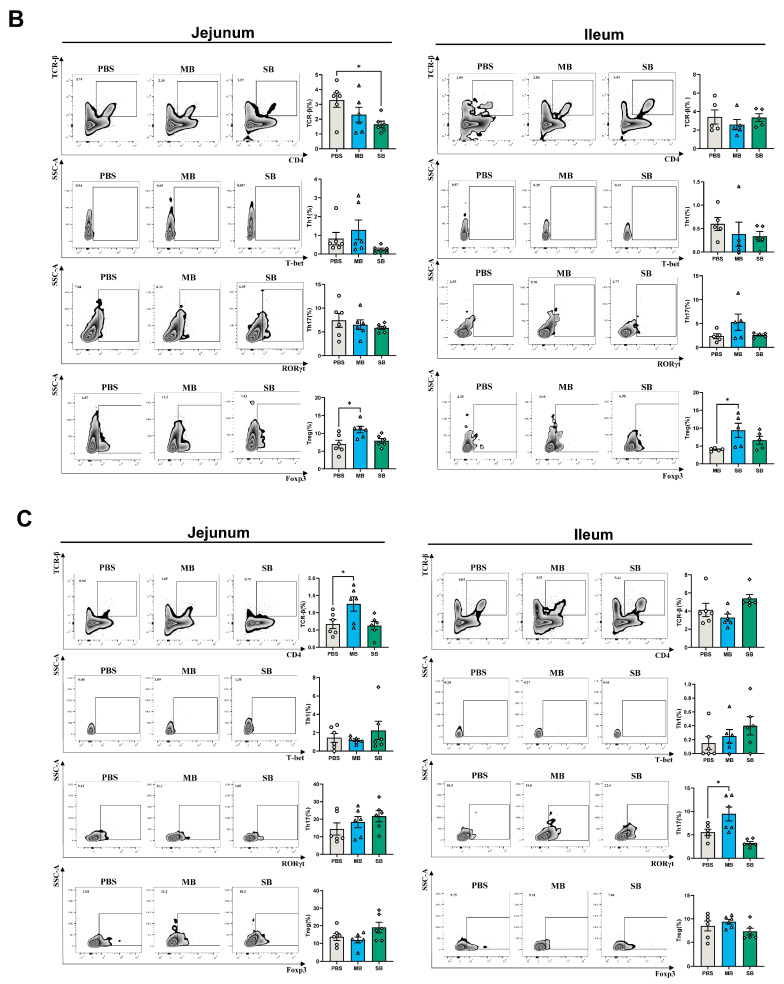
Effect of MB and SB administration on the proportion of immune cells (**A**–**C**), respectively, the effect of MB and SB administration on the proportion of TCR-β, Th1, Th17, and Treg cells in the jejunum and ileum at 1, 7, and 21 days (1 day, n = 3−4; 7 days, n = 5−6; 21 days, n = 6; intestinal samples that failed to isolate small intestinal lamina propria cells were not included in the statistical analysis). * indicate significant difference (*p* < 0.05, respectively).

One day after MB or SB administration, there was no significant effect on the proportions of lamina propria immune cells in the jejunum or ileum (Figure 3A, *p* > 0.05). However, after 7 days of administration (Figure 3B), SB significantly decreased the proportion of TCR-β cells in the jejunum (*p* < 0.05), while no significant difference was found in the ileum (*p* > 0.05). MB and SB had no significant effect on the proportions of Th1 or Th17 cells in the jejunum or ileum (*p* > 0.05). Interestingly, MB significantly increased the proportion of Treg cells in the jejunum and ileum (*p* < 0.05). After 21 days of administration (Figure 3C), MB increased the proportion of TCR-β cells in the jejunum (*p* < 0.05), with no significant difference in the ileum (*p* > 0.05). MB and SB administration had no significant effects on the proportions of Th1 or Treg cells in the jejunum or ileum (*p* > 0.05). Notably, MB administration significantly increased the proportion of Th17 cells in the ileum (*p* < 0.05).

#### 3.4.2. Effect of MB and SB Administration on the Expression of Cytokine mRNA

The expression of intestinal cytokine mRNA is shown in Figure S5. One day after the administration of MB or SB, no significant differences were found in the mRNA expression levels of IL−6, IL−10, IL−17A, IL−22, or TNF−α in the jejunum or ileum (*p* > 0.05). However, MB significantly decreased TGF−β mRNA expression in the jejunum (*p* < 0.05). SB significantly decreased TGF−β mRNA expression in the ileum (*p* < 0.05). After 7 days of treatment, neither MB nor SB had a significant effect on the mRNA expression of IL−6, IL-10, IL-22, or TNF-α in the jejunum (*p* > 0.05). There was a decreasing trend in IL-22 mRNA expression in the jejunum after SB administration (*p* = 0.06). MB and SB had no significant effect on IL-6, IL-10, IL-22, or TGF-β mRNA expression in the ileum (*p* > 0.05). Notably, MB administration significantly decreased TNF-α mRNA expression (*p* < 0.05). SB significantly decreased IL-17A mRNA expression in the ileum (*p* < 0.05). After 21 days of administration, the levels of TGF-β mRNA in the jejunum tended to increase in the SB group (*p* = 0.08). MB and SB significantly decreased IL-6 and IL-17A mRNA expression in the ileum (*p* < 0.05).

### 3.5. Effect of MB Administration on Microbes in the Small Intestinal

#### 3.5.1. Effect of MB Administration on the α and β Diversity of the Ileal Microbiota

**Figure 4:**
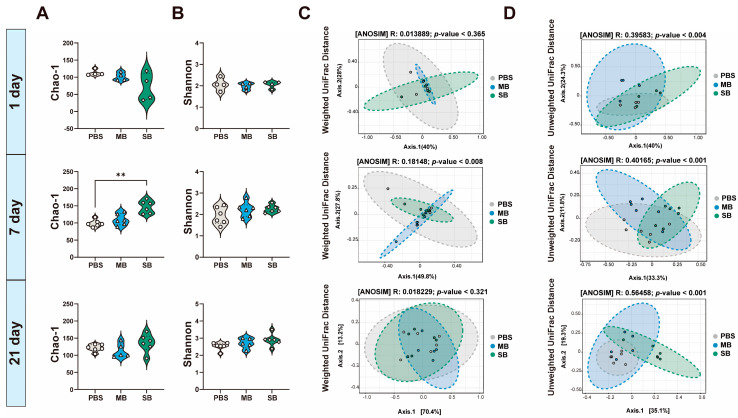
Effects of MB and SB on communities and composition of intestinal microbiota in the ileum 1, 7, and 21 days after intragastric administration. (**A**) Chao1 index of α-diversity. (**B**) Shannon index of α-diversity. (**C**) PCoA plots of β-diversity based on the weighted UniFrac distance. (**D**) PCoA plots of β-diversity based on the unweighted UniFrac. (1 day, n = 4; 7 days n = 6; 21 days n = 5−6; On day 21, one sample from the MB group was excluded due to unqualified sequencing). ** indicate significant difference (*p* < 0.01, respectively).

There was no significant effect on the α diversity of the intestinal microbiota in newly weaned mice 1 day after administration of MB or SB. After 7 days of administration, SB significantly increased the ACE and Chao-1 indices of the microbiota (Figure 4A,B, *p* < 0.05). PCoA was conducted to investigate the effect of MB and SB on the β diversity of the microbiota in the ileum (Figure 4C,D). One day after the administration of MB or SB to the newly weaned mice, the weighted UniFrac distance-based β diversity exhibited no significant difference (ANOSIM, *p* > 0.05). However, differences were observed in the unweighted UniFrac distance-based β diversity. MB administration caused separation of the intestinal microbial communities (ANOSIM, *p* < 0.05). After 7 days of administration, both MB and SB significantly changed the weighted UniFrac distance- and unweighted UniFrac distance-based β diversity, resulting in separation of microbial communities (ANOSIM, *p* < 0.05). After 21 days of intragastric administration of MB or SB, there was no significant difference in the weighted UniFrac distance-based β diversity (ANOSIM, *p* > 0.05). There was a significant difference in the unweighted UniFrac distance-based β diversity of SB-treated mice, leading to the separation of microbial communities (ANOSIM, *p* < 0.05). These results indicate that MB mainly affected the microbial community structure on days 1 and 7 of intragastric administration, while SB affected the microbial diversity and community structure on days 7 and 21 of administration.

#### 3.5.2. Effects of MB Administration on the Abundance of the Ileal Microbiota Constituents

**Figure 5:**
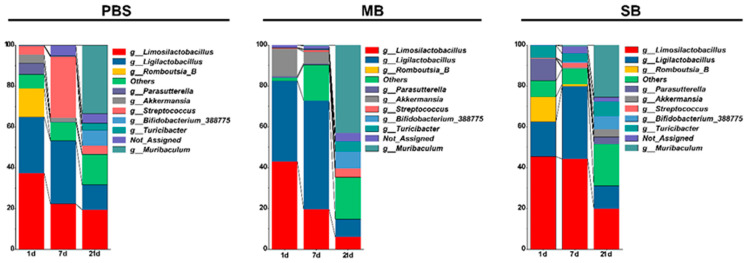
Abundance of intestinal microbiota in the ileum on 1, 7, and 21 days following intragastric administration with MB and SB.

To further investigate the alterations in the ileal microbiota caused by MB and SB administration, we compared the taxonomy of the top 10 most abundant microbiota constituents at the genus level (Figure 5). The Kruskal–Wallis test showed significant effects of MB and SB on the microbiota composition at the genus level (Tables S2–S4). In the PBS intragastric administration group, from the beginning of weaning to maturity (from 1 to 21 days of intragastric administration), the gradually depleted microbiota constituents included *Limosilactobacillus* and *Akkermansia*; the gradually enriched microbiota constituents included *Muribaculum*, *Turicibacter*, and *Bifidobacterium*; the microbiota constituents that were sharply depleted upon completion of the weaning response included *Parasutterella* and *Romboutsia*; and the microbiota constituents that were initially enriched and then depleted from the start of weaning to maturity included *Ligilactobacillus* and *Streptococcus*. The Kruskal–Wallis test revealed the effect of intragastric MB and SB administration on the abundance of microbiota constituents at the genus level. One day after MB administration, MB reduced *Romboutsia_B* abundance by 13.42% (*p* < 0.05). After 7 days of treatment, MB increased *Muribaculum* abundance by 0.44% (*p* < 0.05). SB increased *Limosilactobacillus*, *Muribaculum*, and *Turicibacter* abundance by 22.45%, 0.79%, and 3.45%, respectively (*p* < 0.05). After 21 days of administration, MB and SB did not significantly affect the relative abundance of the top 10 genera in the microbiota.

#### 3.5.3. Effect of MB Administration on the Maturation of the Gut Microbiota

**Figure 6:**
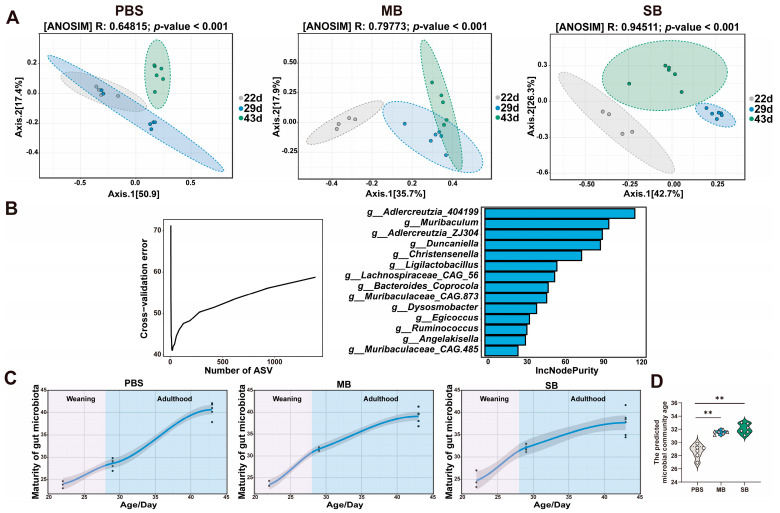
Effect of MB and SB administration on the maturation of the ileum microbiota. (**A**) β-diversity based on the unweighted UniFrac results at different intragastric administration. (**B**) Prediction of the microbiota at the genus level after 100 iterations of tenfold cross-validation and the top 14 prediction genera. (**C**) The microbiota maturation curve. (**D**) The statistical difference analysis of the predicted microbiota age on the 7 days of the intragastric administration experiment. ** indicate significant difference (*p* < 0.01, respectively).

We used PCoA to analyze the changes in microbial community structure based on unweighted UniFrac distances at different ages among the three groups of animals (Figure 6A). Subsequently, we utilized a random forest algorithm to predict the microbial ages of the microbiota from the mice in the MB- and SB-treated groups, using the PBS-treated group as the training set (Figure 6B,C). The PCoA results showed that in the PBS group, the microbial community on day 21 was separated from the microbial community on day 1 and day 7, and there was no significant separation between the microbial communities on day 1 and day 7 (ANOSIM, *p* < 0.05). Similar findings have been reported in previous studies [7]. Within the MB administration group, the microbial community observed on day 7 overlapped with that on day 21 and was distinctly segregated from the community observed after 1 day of administration. In the SB administration group, the microbial community sampled was separated across three time intervals, and the microbial community at 7 days of administration was closer to that at 21 days of administration. These results suggest that MB and SB administration may facilitate the convergence of the microbial community structure toward the microbial community structure associated with physical maturity. To further validate this hypothesis, we used the random forest algorithm. We performed 100 iterations with tenfold cross-validation to determine the number of genera required to construct the model (Figure 6B,C); the model consisted of 14 genera. The accuracy of the model predictions increased slightly when different numbers of bacterial genera were used to construct the model. This model was used to predict the age of the ileum microbiota in mice 22–43 days of age (70.66% variation explained). Statistical analysis of the microbial ages predicted by the model showed that on day 7 of MB and SB administration, the predicted microbial ages of the mice in the MB and SB groups were significantly greater than that of the mice in the PBS group (*p* < 0.05) (Figure 6D). The microbial maturation curve also showed that the ileum microbiota reached a plateau more rapidly after MB and SB administration than after PBS administration (Figure 6C). This finding underscores the notion that MB and SB administration can facilitate the maturation of the ileum microbiota in mice.

#### 3.5.4. Effect of MB Administration on the Ileal Microbial Co-Occurrence Network

**Figure 7:**
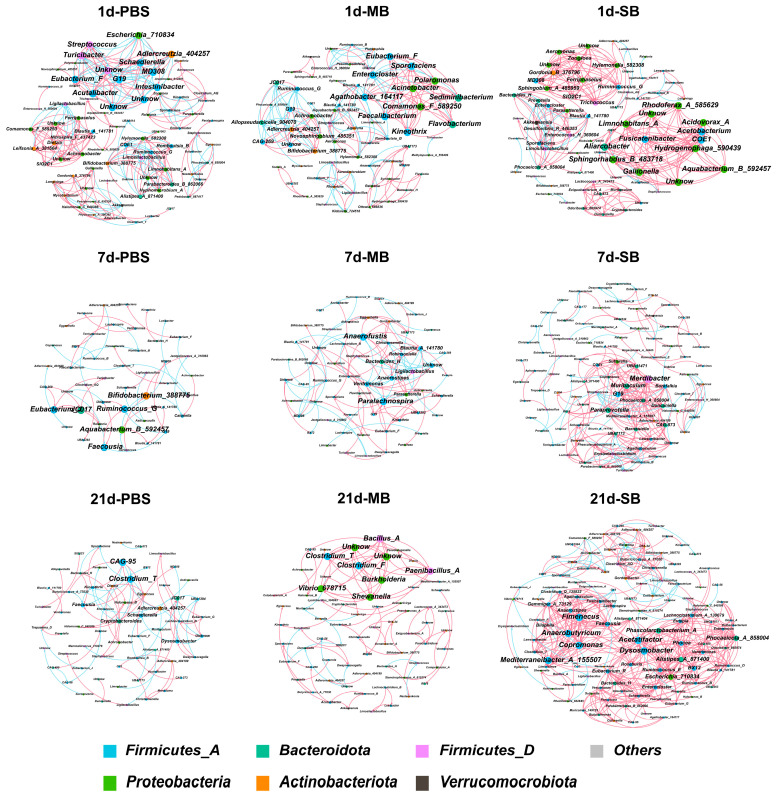
The results of the co-occurrence network analysis of MB and SB intragastric administration.

As shown in Figure 7 and Table S5, microbial co-occurrence network analysis elucidated the network structure and interplay among the ileal microbiota. All three experimental groups exhibited a pattern of decreasing complexity followed by increasing complexity of the microbiota co-occurrence network over time. In the PBS group, from the beginning to the end of intragastric administration (day 1 to day 21), the total nodes, total edges, and average degree first decreased and then increased. In the MB administration group, the total nodes, proportion of positive correlations, and the clustering coefficient showed the same trend. This pattern was also observed in the SB administration group in terms of total edges, average degree, proportion of positive correlations, and clustering coefficient. Subsequently, we performed a comparative analysis of the network complexity among the groups at the same time point. Notably, after 1 day of intragastric administration of MB and SB, the complexity of the microbiota network was slightly reduced, which was characterized by a decrease in the total number of nodes and edges. However, both MB and SB significantly increased the overall complexity of the ileal microbiota network after 7 and 21 days of administration. This elevated complexity was reflected in the increase in the total number of nodes and edges, higher average degree, and increase in the proportion of positive correlations. Notably, the increase in network complexity was more pronounced in the SB group than in the MB group. These results suggest that MB and SB accelerate alterations in the mouse ileal microbiota network and increase the complexity of the co-occurrence network.

### 3.6. The Effect of MB on Intestinal Th17 Cell Differentiation May Be Attributed to Changes in the Intestinal Microbiota

#### 3.6.1. Relative Abundance and Quantification of Segmented Filamentous Bacteria (SFBs) in the Ileum

**Figure 8:**
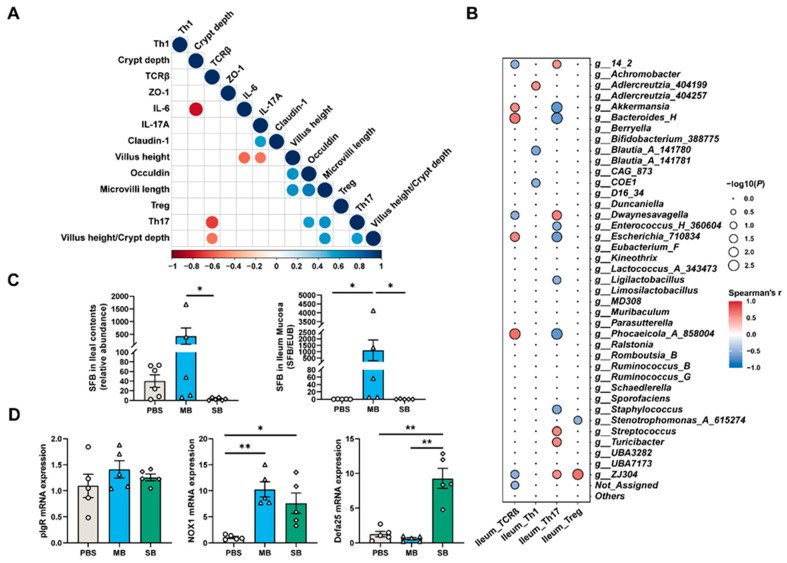
MB increases the proportion of Th17 cells in the ileum by increasing the abundance of adhesive SFB bacteria. (**A**) Indices related to intestinal health and ileal immune cells based on Spearman correlation analysis. (**B**) Spearman correlation analysis of the ileum microbiota at the top 40 genera and the proportion of ileum immune cells. (**C**) Relative abundance (intestinal contents) and relative quantification (intestinal mucosa) of SFB (n = 5). (**D**) mRNA expression of the downstream pathway of IL-17R (n = 5). * and ** indicate significant difference (*p* < 0.05 and *p* < 0.01, respectively).

As depicted in Figure 8A, the proportion of Th17 cells correlated positively with occludin mRNA expression and microvillus length. LEfSe analysis revealed that *Dwaynesavagella* (also known as *Candidatus Arthromitus*) was a biomarker in the MB group after 21 days of treatment (Figure S6). The literature suggests that SFB monocolonization can promote the development of Th17 cells in the gut, and a lack of SFBs in the intestine could lead to Th17 cell defects [35,36]. Regarding the top 40 genera of the ileal microbiota and their correlation with ileal immune cells, we found a strong positive correlation between the relative abundance of *Dwaynesavagella* and the proportion of Th17 cells (Figure 8B). We retrieved the microbial sequence of *Dwaynesavagella* from the sequencing results and found the highest sequence similarity with SFBs in the NCBI database, as indicated in Table S6. Differential analysis of the relative abundance of *Dwaynesavagella* showed that the relative abundance in the MB-treated group was significantly greater than that in the SB-treated group (*p* < 0.05). Subsequently, we quantified the relative abundance of *Dwaynesavagella* in the intestinal mucosa and found that the relative abundance of *Dwaynesavagella* in the intestinal mucosa in the MB-treated group was significantly greater than that in both the PBS- and SB-treated groups (Figure 8C, *p* < 0.05). Therefore, we postulate that the promotion of Th17 cell development in the ileum by MB may be associated with increased SFB colonization in the ileal mucosa.

#### 3.6.2. Effect of MB Administration on the Expression of Genes Downstream of the IL-17 Receptor

Finally, we validated the downstream genes regulated by the IL-17 receptor in the ileum (Figure 8D) and found that the administration of MB increased NOX1 mRNA expression (*p* < 0.05). However, pIgR and Defa25 mRNA expression was not significantly affected (*p* > 0.05). Notably, SB administration significantly increased the expression of Defa25 mRNA (*p* < 0.05).

## 4. Discussion

The weaning stage is a pivotal phase in an animal’s growth and development. Any impairment in the development of intestinal barrier function during this period could have profound implications [7,11]. In this study, we primarily investigated the changes in indices related to the small intestinal microbiota and intestinal health of weaned mice at different times, aiming to explore the impact of MB on the gut microbiota and intestinal health of weaned mice. The experimental results indicate that MB can regulate microbiota development during the weaning period and affect the abundance of related gut microbiota constituents during the maturation period, benefiting the host’s intestinal health.

MB, as a butyric acid derivative, seems to be quickly utilized by the body. In the MB group, the peak butyric acid concentration in the plasma of the mice reached 0.82 mM at 0.5 h after administration (Figure S1). Previous studies have reported similar results [37,38]. The MB molecule contains ester bonds that can be hydrolyzed by carboxylesterase in tissues or serum [39]. Compared with SB, MB administration significantly increased serum and ileum carboxylesterase activity at 1 h after administration (Figure S2).

MB and SB administration for 7 days improved intestinal mucus layer function. After 21 days of intragastric administration, MB did not increase the number of goblet cells or MUC2 mRNA expression in the small intestine. During the maturation stage, the positive effect of MB on the function of the small intestinal mucus layer in the mice was not as pronounced as that of SB. MB and SB administration on day 1 decreased claudin-1 mRNA expression in the jejunum and ileum. On day 7, occludin mRNA expression was also reduced. A previous study showed that claudin-1 expression in the jejunum of mice decreases gradually as the mice age, along with normal physiological development [40]. This result was also observed in rabbits and mice [41,42]. On days 7 and 21 of intragastric administration, MB exerted a beneficial influence on the intestinal brush border structure, resulting in an increase in the microvillus length. In addition to the observation of the microstructure of the intestinal epithelial cells (IECs), it was noted that MB and SB had no negative effects on the microstructure of the microvilli or tight junctions. Furthermore, after 21 days of intragastric administration, MB increased occludin and ZO-1 mRNA expression. Therefore, the reduction in tight junction-related mRNA expression detected on days 1 and 7 in the present study may be attributed to the accelerated development of the intestinal tight junction structure induced by MB and SB.

A previous study indicated that during the weaning period, there was an initial increase in TNF-α and IFN-γ levels, which stabilized by 28–35 days of age, showing a bell-shaped trend [11]. Here, MB and SB administration on day 1 did not affect TNF-α mRNA expression. However, on day 7 after administration, which corresponds to 29 days of age for the mice, MB significantly decreased TNF-α expression in the ileum. Thus, during the early stages of weaning, MB did not alter the weaning response. However, MB may have accelerated the return of TNF-α levels to a stable state.

In this study, we observed that MB and SB have distinct regulatory effects on the development of intestinal immune cells. The development of Treg cells in the small intestine is initiated with antigen stimulation and is comparable to that of adult mice at 4 weeks of age [6]. A previous study showed that SCFAs promote the differentiation of Treg cells in the colon of SPF mice but have no significant effect on the differentiation of Treg cells in the small intestine [15]. The SB administration group presented similar results. However, MB significantly increased the proportion of Treg cells on day 7 of administration. MB significantly increased the proportion of Th17 cells in the ileum after 21 days of treatment. To determine the underlying mechanism, we measured cytokine mRNA expression, investigating whether the changes in cell proportions resulted from MB-induced immunomodulation. The findings indicated that after 21 days of intragastric administration, MB and SB decreased the expression of IL-6 and IL-17 mRNA in mice. This finding suggested that during the maturation of the mice, MB and SB modulated the immune response, further reducing the level of intestinal proinflammatory cytokines. However, the levels of cytokines related to the regulation of Treg cell differentiation, such as TGF-β, did not change significantly on day 7 [6]. Given these findings, we focused on the gut microbiota.

In vitro experiments have demonstrated that MB affects the proliferation of *Clostridium perfringens* and regulates the cecal microbiota of rats [19,43]. MB altered the microbial community structure in the small intestine in our study, in both the short term and long term. Treg cells in the small intestine are mainly divided into two categories: Helios^+^ Treg cells and RORγt^+^ Treg cells [44]. Intestinal microbial antigens can modulate RORγt^+^ Treg cells in the small intestine [45]. In our study, differential analysis results indicated a significant increase in *Lachnospiraceae* abundance in the MB administration group compared to that in the other treatment groups. This finding was further supported by LEfSe analysis, which revealed *Lachnospiraceae bacterium_COE1* as a biomarker in the MB-treated group. This finding coincides with the increase in the proportion of Treg cells observed on day 7 in the MB-treated group. Interestingly, *Lachnospiraceae bacterium_COE1* appeared to be a biomarker in the PBS administration group but only on day 21. Previous studies have confirmed the role of *Lachnospiraceae* within the microbial community in regulating the proportion of Treg cells in the large intestine without affecting that in the small intestine [46,47]. However, research conducted by Lubin et al. indicated that during the weaning period, specific microbial species can modulate the proportion of RORγt^+^ Treg cells in the small intestine [7]. The specific reason for the increase in the proportion of Treg cells in the small intestine after 7 days of intragastric MB administration remains unclear. However, it can be postulated that this finding is likely associated with the composition of specific intestinal microbiota constituents.

SFBs colonize the intestines of various species, including humans and mice, primarily in the ileum [48]. Their colonization was related to a decrease in the abundance of pathogenic microorganisms [49]. An increase in SFB abundance further increases the proportion of Th17 cells in the ileum of SPF mice [36], and this regulatory effect on intestinal Th17 cells has also been observed with nonadhesive SFBs [50]. In our study, MB increased the proportion of ileal Th17 cells by enriching both adhesive and nonadhesive SFBs on day 21. Our analysis of intestinal cytokine expression indicated that the increase in the proportion of Th17 cells did not lead to an increase in the expression of the associated proinflammatory cytokines in the intestine. The levels of Th17 cells correlate positively with the enhancement of tight junctions and brush border structures. Furthermore, research indicates that commensal-induced Th17 cells contribute to the functionality of the intestinal barrier and host defense. Conversely, the downstream regulation of IL-17R on cells contributes to increased expression of host immune function genes, such as NOX1. This finding suggested that the MB-induced increase in Th17 cell levels may benefit host defense.

This study also has several limitations. For instance, the experimental results were not verified in female mice. Differences in carboxylesterase levels between human and mouse plasma could impact the degradation and utilization of MB [51]. Future research could employ encapsulation slow-release technology. This approach would allow MB to bypass digestion in the intestine and target specific intestinal sites. Despite these limitations, MB demonstrated excellent effects as a nutritional dietary supplement. It effectively regulates the intestinal barrier and immune system and could serve as a substitute for TB or other butyric acid products.

## 5. Conclusions

MB can enhance the development of the intestinal physical barrier and improve intestinal morphology. MB promotes the maturation of Treg cells and the intestinal microbiota in weaned mice. During physiological maturation, it may be possible to promote intestinal health by modulating the gut microbiota to increase the proportion of Th17 cells in the ileum. As a dietary supplement, MB exerts effects on the gut that may outweigh its role as a butyric acid supplier. Given these properties, MB shows potential as a nutritional supplement for use in young animals.

## Data Availability

The data used to support the findings of this study are available from the corresponding author upon request.

## Supplementary Materials

The following supporting information can be downloaded at: https://www.mdpi.com/article/10.3390/nu16132052/s1, Figure S1: The concentration of butyric acid in the plasma following the MB administration. (n = 3), Figure S2: The activity of carboxylesterases in the serum, liver, and small intestinal following the MB administration. (n = 3), Figure S3: Flow cytometry gating strategy, Figure S4: Effect on the intestinal morphological structure of mice on 1, 7, and 21 days after MB and SB administration, Figure S5: Effect of MB and SB administration on the expression of cytokine mRNA in the jejunum and ileum at 1, 7, and 21 days (1 day, n = 3; 7 and 21 days, n = 5), Figure S6: Effect of MB administration on the biomarkers of ileal microbiota in mice 1, 7, and 21 days after administration, Table S1: Primer Information, Table S2: Statistical analysis of the abundance of top 10 bacterial relative abundance proportion at the genus level after 1 day with MB and SB administration, Table S3: Statistical analysis of the abundance of top 10 bacterial relative abundance proportion at the genus level after 7 days with MB and SB administration, Table S4: Statistical analysis of the abundance of top 10 bacterial relative abundance proportion at the genus level after 21 days with MB and SB administration, Table S5: The co-occurrence network properties at genus level, Table S6: NCBI BLAST results for the most significant ASV sequence of *Dwaynesavagella* in the ileum of mice.
