# Supplementary material for: Monobutyrin Can Regulate the Gut Microbiota, Which Is Beneficial for the Development of Intestinal Barrier Function and Intestinal Health in Weaned Mice
Source: Nutrients. 2024 Jun 27;16(13):2052. doi: 10.3390/nu16132052 (PMC11243092; doi:10.3390/nu16132052)
Supplement: Supplementary file 1 [file nutrients-16-02052-s001.zip › -ú+s-MB-supplementary files 6.27.pdf]

nutrients

Methods and Materials：

Health monitoring reports

Test Item	Test Method	Tested	Results
Mouse Hepatitis Virus (MHV) Ab	ELISA-IgG	2	Negative
Sendai Virus (SV) Ab	ELISA-IgG	2	Negative
Pneumonia Virus of Mouse (PVM) Ab	ELISA-IgG	2	Negative
Minute Virus of Mice (MVM) Ab	ELISA-IgG	2	Negative
Salmonella spp.	Culture Method	2	Negative
Clostridium piliforme Ab	ELISA-IgG	2	Negative
Mycoplasma spp. Ab	ELISA-IgG	2	Negative
Corynebacterium kutscheri	Culture Method	2	Negative
Pasteurella pneumotropica	Culture Method	2	Negative
Pneumonia Virus of Mouse (PVM) Ab	Culture Method	2	Negative
Pseudomonas aeruginosa	Culture Method	2	Negative
Ectoparasites	Microscopy Test	2	Negative
Toxoplasma gondii (T. gondii) Ab	ELISA-IgG	2	Negative
Helminths	Microscopy Test	2	Negative
Flagellates	Microscopy Test	2	Negative
Ciliates	Microscopy Test	2	Negative
Helicobacter spp.	PCR	2	Negative
Corynebacterium bovis (CBOV)	Culture Method	2	Negative
Mouse norovirus (MNV) Ab	ELISA-IgG	2	Negative

Animal Experiment (Plasma butyric acid concentration)

After one week adaptation and fasting for 12 hours, three healthy 6-week-old male C57BL/6 mice (22.01±0.70) were intragastrical administered 1 g/kg BW of MB. plasma samples were collected from the retro-orbital venous plexus at 0, 0.5, 1.5, 3, and 6 hours after intragastric administration. Post the administration of isoflurane-induced anesthesia to the mice, approximately 50 μL of plasma was collected into anticoagulant tubes containing sodium heparin. Following the completion of all plasma collection procedures, the mice were euthanized using pentobarbital sodium. Forty μL of plasma sample was mixed with saturated sodium sulfate solution acidified with 0.1 M hydrochloric acid. The mixture was vortexed and allowed to stand at room temperature for 30 minutes. After centrifugation at 4000 rpm for 15 minutes at room temperature, the upper liquid was collected and transferred to 1.5 mL headspace vials for subsequent headspace gas chromatography analysis.

Plasma butyric acid concentration Detection by Headspace Gas Chromatography

The quantification of butyric acid content in plasma samples was performed using gas chromatography in headspace mode with the following specifications: gas chromatograph: Agilent 7890B (California, USA); sample introduction: 1.5-mL tight gas syringe; column: 19091G-B113, 30 m × 320 μm × 0.25 μm (Agilent, California, USA); oven temperature program: initial temperature: 60 °C for 3 minutes, heating rate: 20 °C per minute until reaching 180 °C; carrier gas: nitrogen, septum purge flow rate: 3.0 mL/min, split ratio: 20:1, split flow rate: 30 mL/min; flame ionization detector (FID) parameters: detector temperature: 250 °C; air flow rate: 350 mL/min; hydrogen flow rate: 35 mL/min.

Animal Experiment (Carboxylesterase Activity Analysis)

A total of 27 healthy 6-week-old male C57BL/6 mice (22.69±2.21) were used in this experiment. The source of mice and the experimental management conditions followed the description provided in Animal Experiment 1. After a one-week adaptation period, the mice with similar BW were stratified into 3 groups, and intragastrical administered 0.4 mL of PBS, MB, SB, or tributyrin (TB) at the dosage of 1 g/kg BW. The mice were euthanized at 0, 1, and 6 hours after intragastric administration. The serum samples and intestinal segment samples were collected following the procedures described in 2.2 Animals and Experimental Protocol.

Carboxylesterase Activity Analysis

The carboxylesterase activities in the serum, liver, duodenum, jejunum, and ileum were determined using carboxylesterase assay kits (Sangon Biotech, Shanghai, China). The serum samples were directly assayed after a 5-fold dilution with distilled water. The tissue samples were homogenized in a 1:15 mass-to-volume ratio with distilled water, followed by centrifugation at 1200 rpm at 4 °C for 15 minutes. The supernatant was collected for carboxylesterase activity analysis in accordance with the kit test instructions.

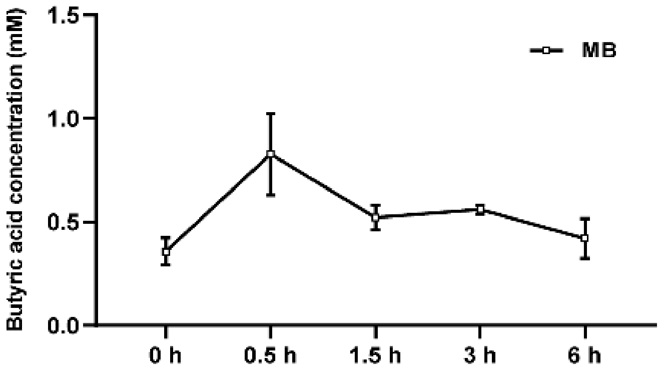

Figure S1. The concentration of butyric acid in the plasma following the MB administration. (n=3).

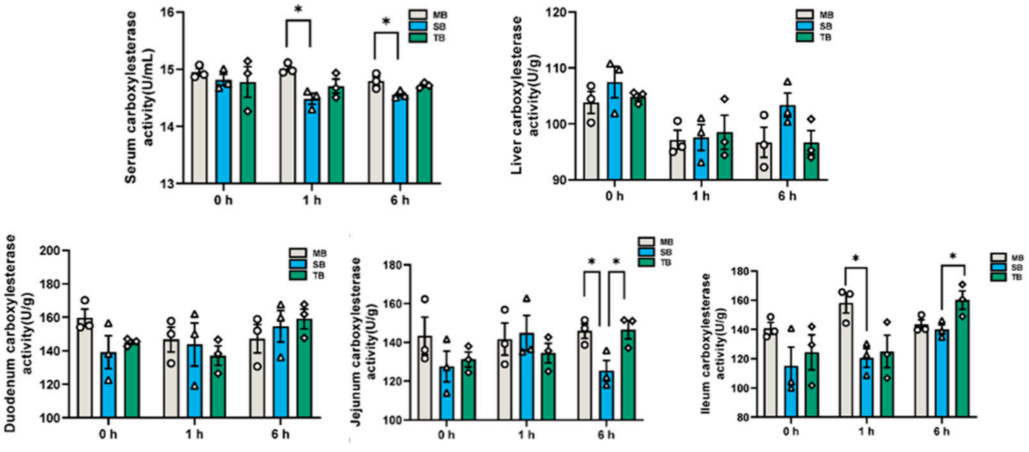

Figure S2. The activity of carboxylesterases in the serum, liver, and small intestinal following the MB administration. (n=3).

Table S1. Primer Information.

Genes	Forward Primer (5′-3′)	Reverse Primer (5′-3′)
IL-6	GAGAGGAGACTTCACAGAGGATACC	TCATTTCCACGATTTCCCAGAGAAC
IL-10	ATGAGTTTTTCCCTTATGGGGAC	GCTGGAAGTTGGACACCTCAA
IL-17A	TCAGCGTGTCCAAACACTGAG	CGCCAAGGGAGTTAAAGACTT
IL-22	ATGAGTTTTTCCCTTATGGGGAC	GCTGGAAGTTGGACACCTCAA
TNF-α	GCCTCTTCTCATTCCTGCTTGTGG	GTGGTTTGTGAGTGTGAGGGTCTG
Tgf-β	GCTGAACCAAGGAGACGGAATAC	CGTGGAGTTTGTTATCTTTGCTGTC
Claudin-1	CCTGGCTTCTCTGGGATGGATC	CTGAGCGGTCACGATGTTGTC
Occludin	TTGAAAGTCCACCTCCTTACAGA	CCGGATAAAAAGAGTACGCTGG
ZO-1	GCCGCTAAGAGCACAGCAA	TCCCCACTCTGAAAATGAGGA
MUC2	GCTGACGAGTGGTTGGTGAATG	GATGAGGTGGCAGACAGGAGAC
NOX1	AAATTCCAGCGTGCCGACAAC	TTGCCTAATTCCTCCATCTCTTGTTC
PIgR	GCTCTACTTGTTCACGCTCTTGG	CGCCTTCTATACTACTCACCTCCTG
Defa25	GCAAAAGAAGAGAACGCCTGAATG	CAGCAGCACCAGAGCATGTAC
GAPDH	AGGTCGGTGTGAACGGATTTG	TGTAGACCATGTAGTTGAGGTCA
SFB	TGAGCGGAGATATATGGAGC	CATGCAACTATATAGCTATATGCGG
EUB	ACTCCTACGGGAGGCAGCAGT	ATTACCGCGGCTGCTGGC

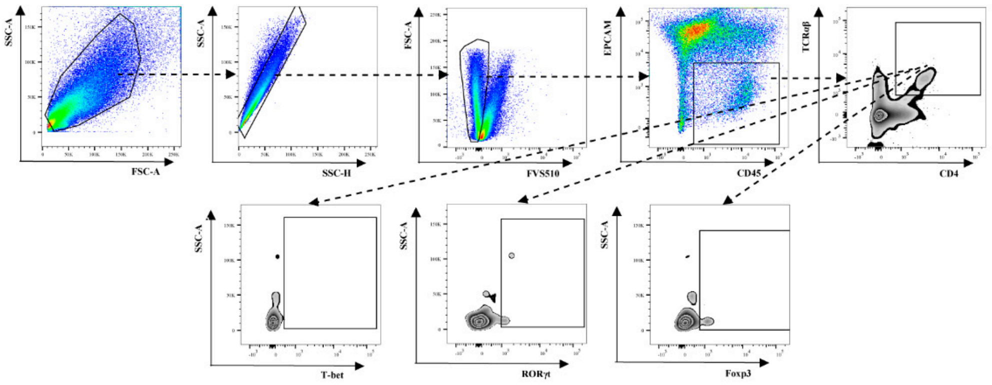

Figure S3. Flow cytometry gating strategy.
