# Supplementary figures and images for: Monobutyrin Can Regulate the Gut Microbiota, Which Is Beneficial for the Development of Intestinal Barrier Function and Intestinal Health in Weaned Mice
Source: Nutrients. 2024 Jun 27;16(13):2052. doi: 10.3390/nu16132052 (PMC11243092; doi:10.3390/nu16132052)

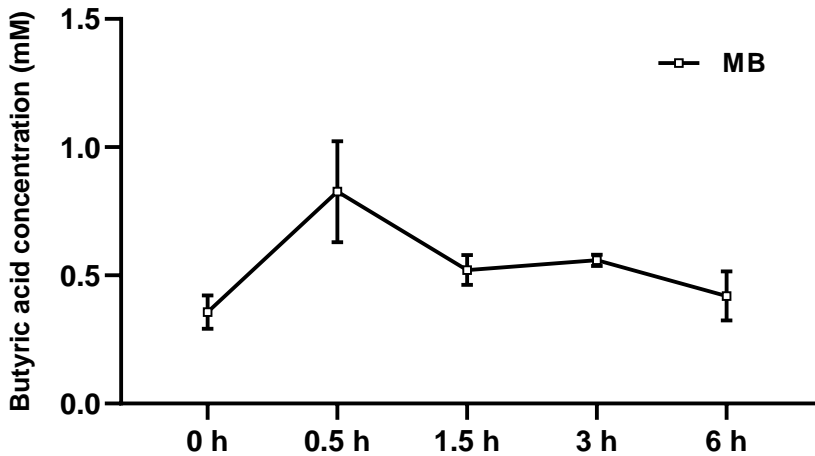

Supplement: Supplementary file 1 [file nutrients-16-02052-s001.zip › figure S1.pdf]

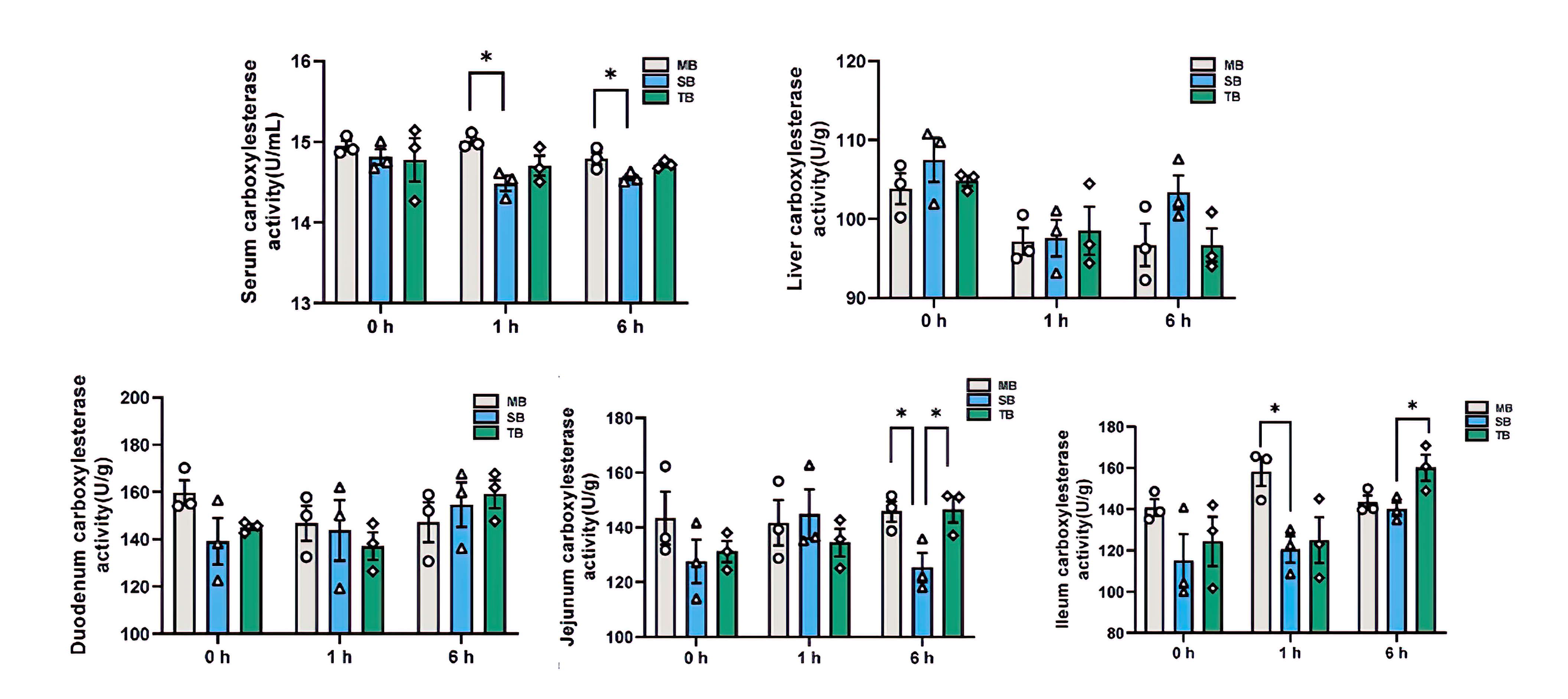

Supplement: Supplementary file 1 [file nutrients-16-02052-s001.zip › figure S2.png]

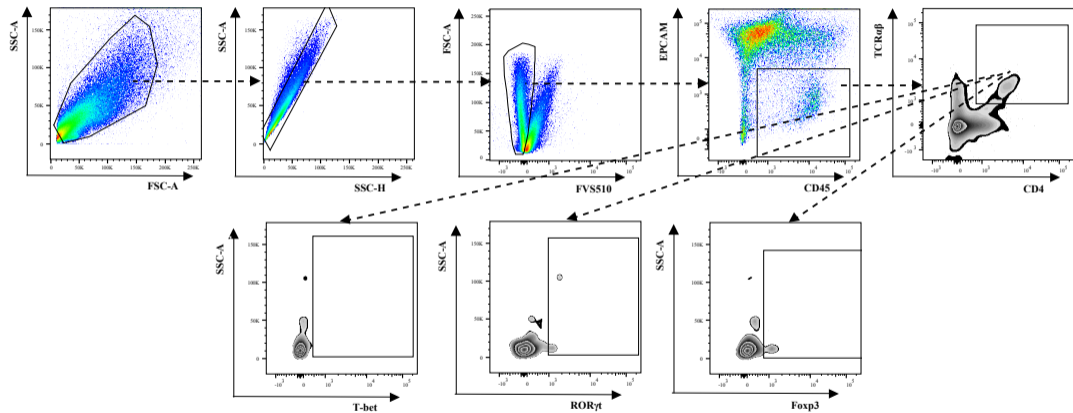

Supplement: Supplementary file 1 [file nutrients-16-02052-s001.zip › figure S3.pdf]

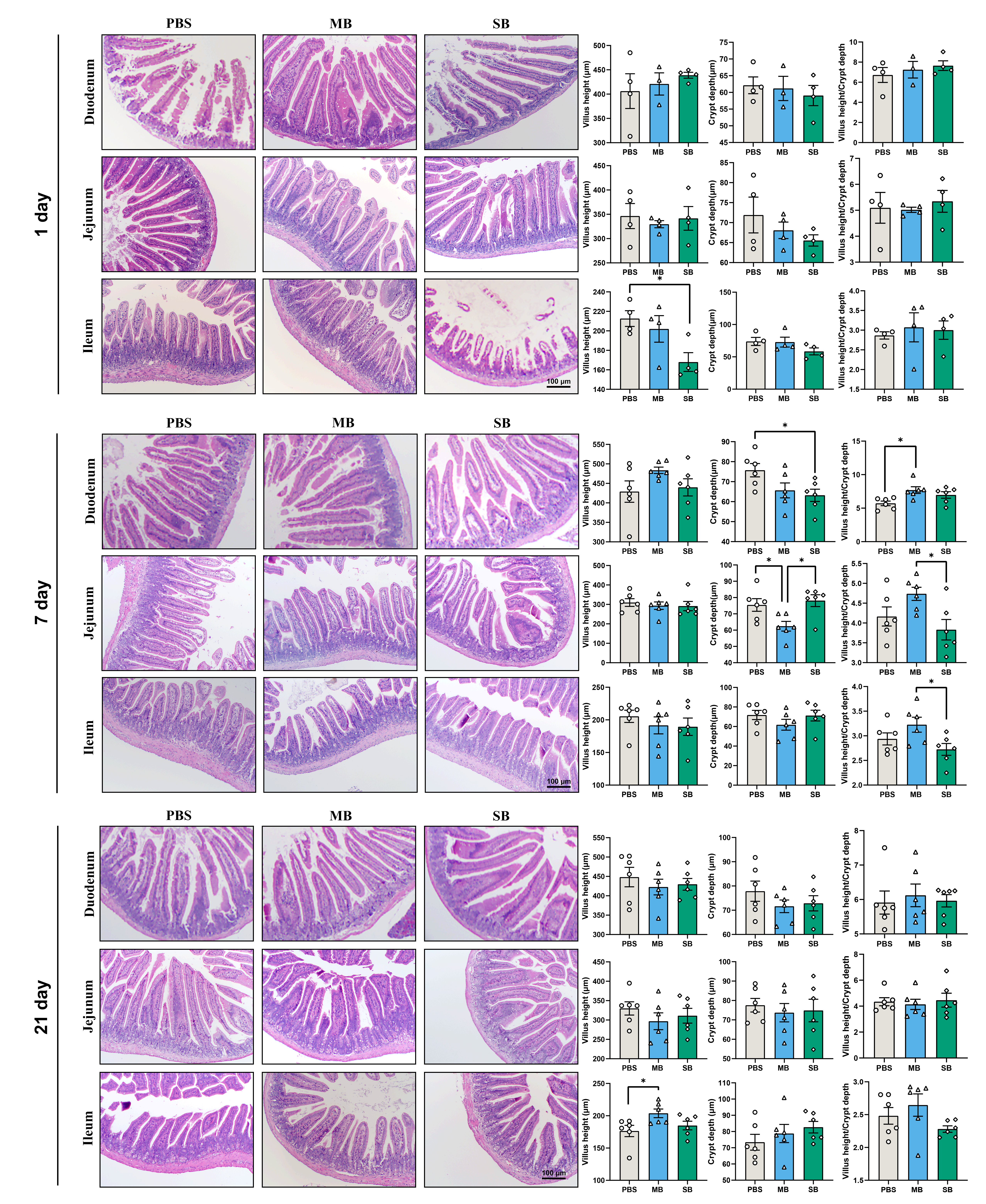

Supplement: Supplementary file 1 [file nutrients-16-02052-s001.zip › figure S4.tif]

**A**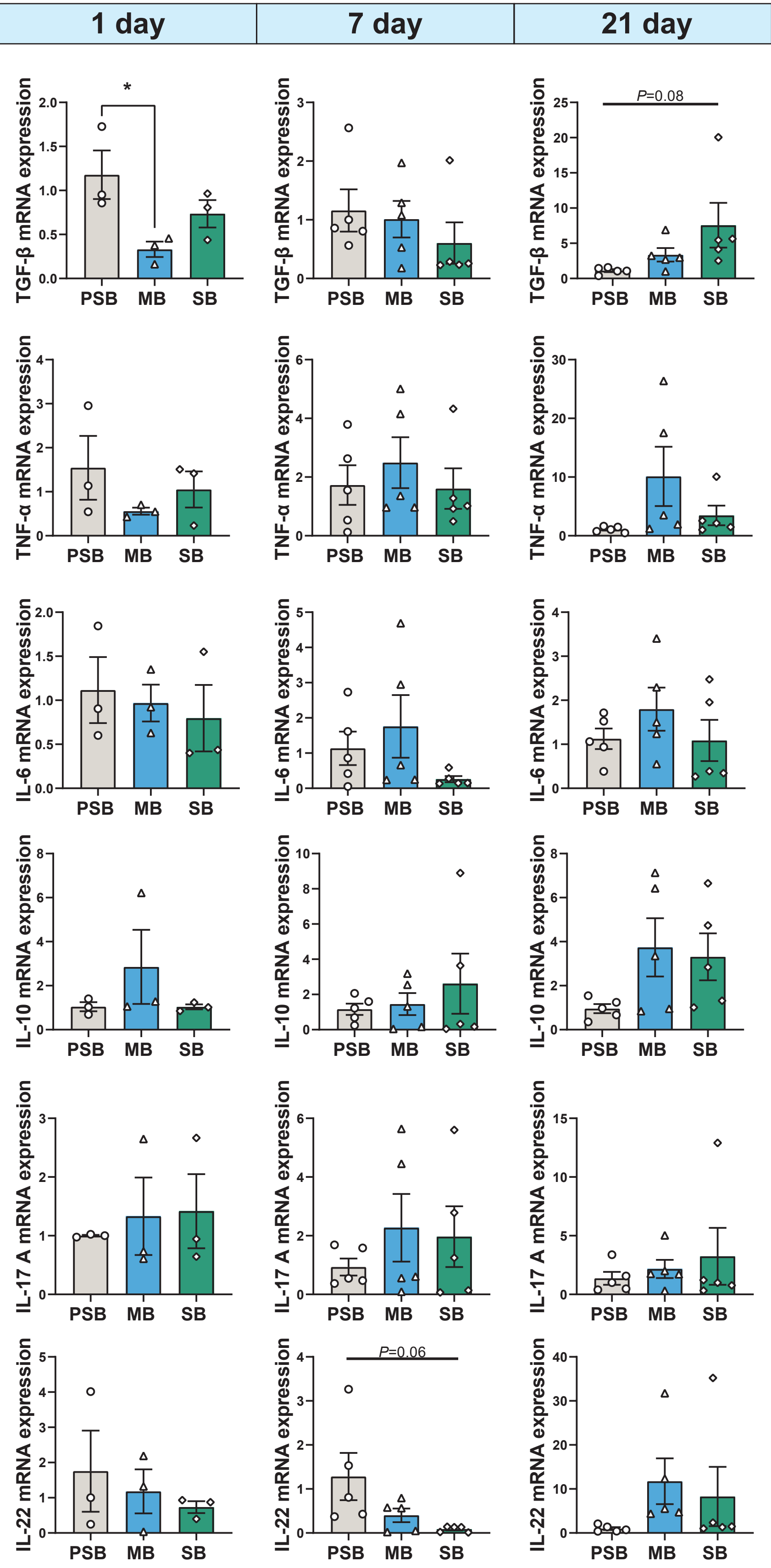**Jejunum****B**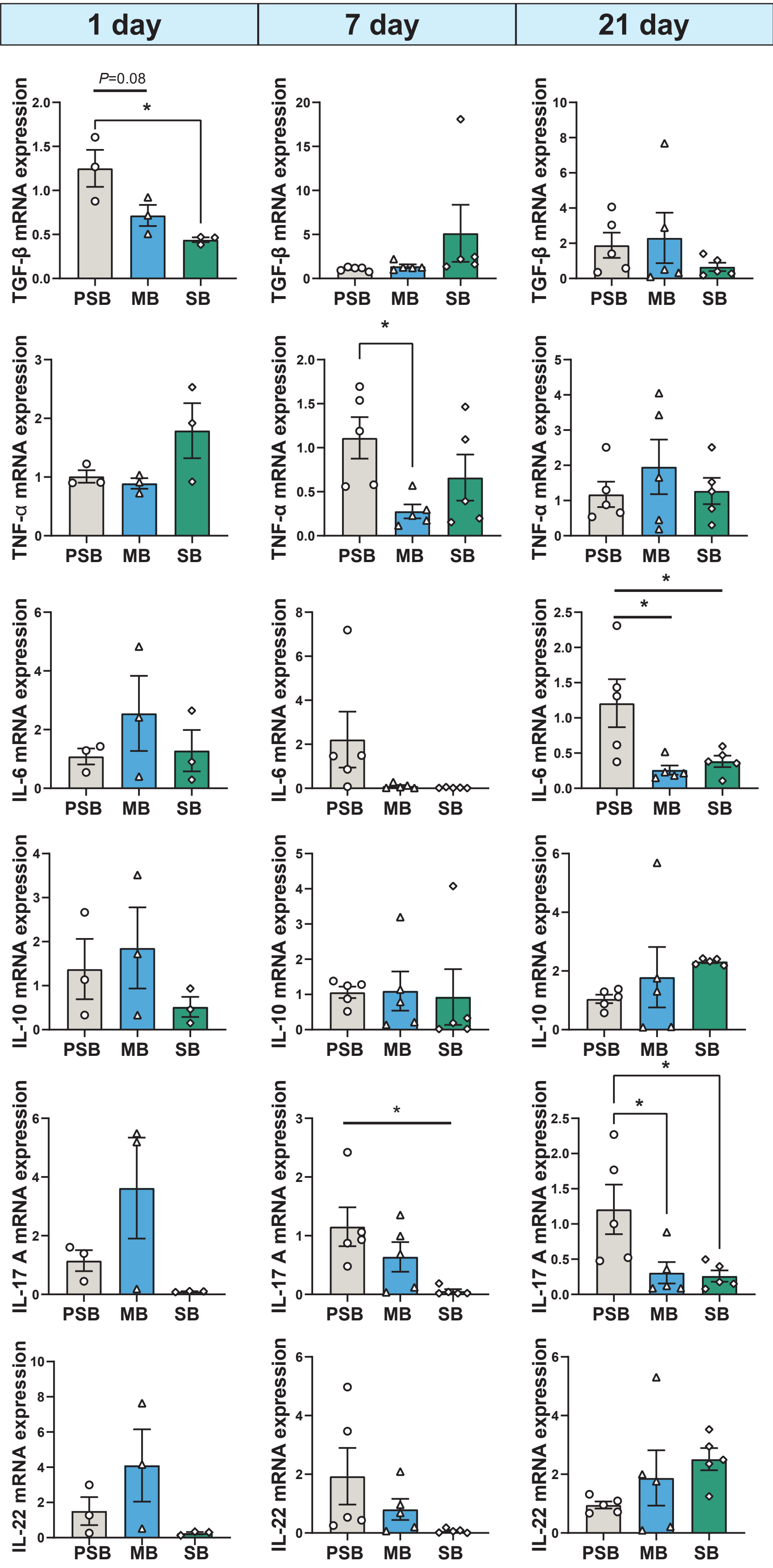**Ileum**

Supplement: Supplementary file 1 [file nutrients-16-02052-s001.zip › figure S5.pdf]

A

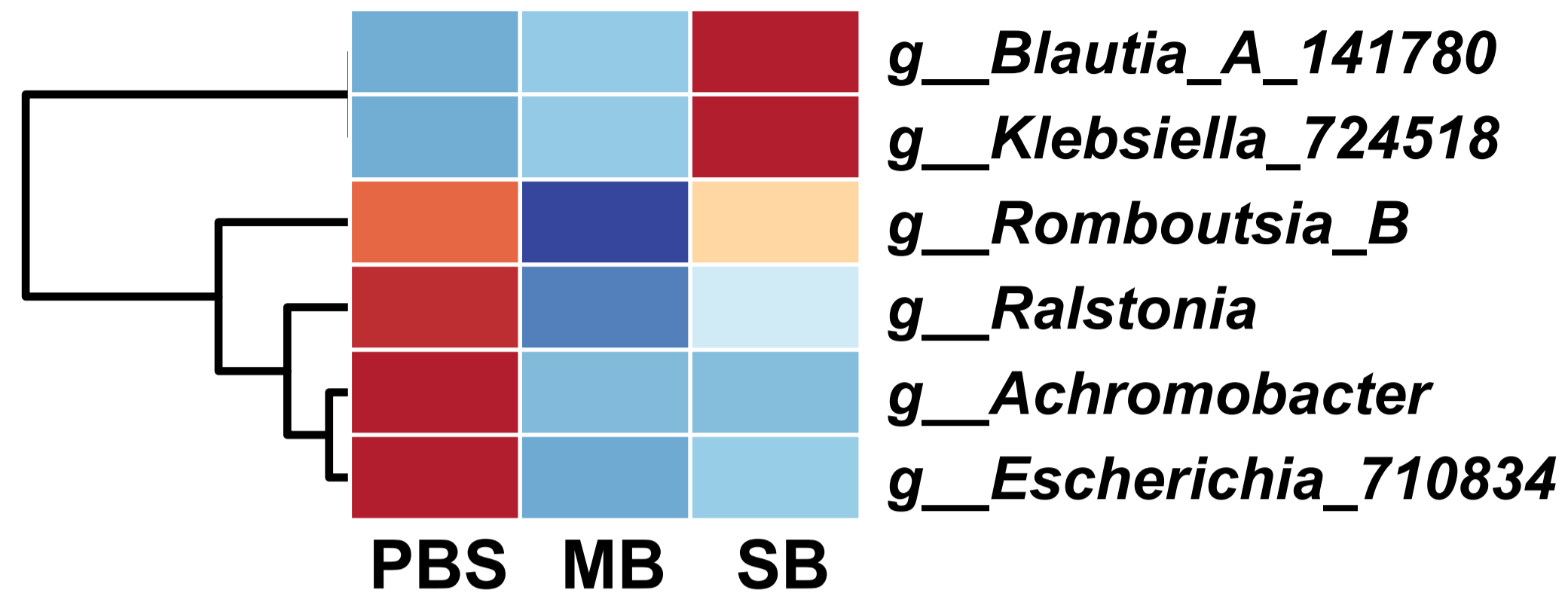

B

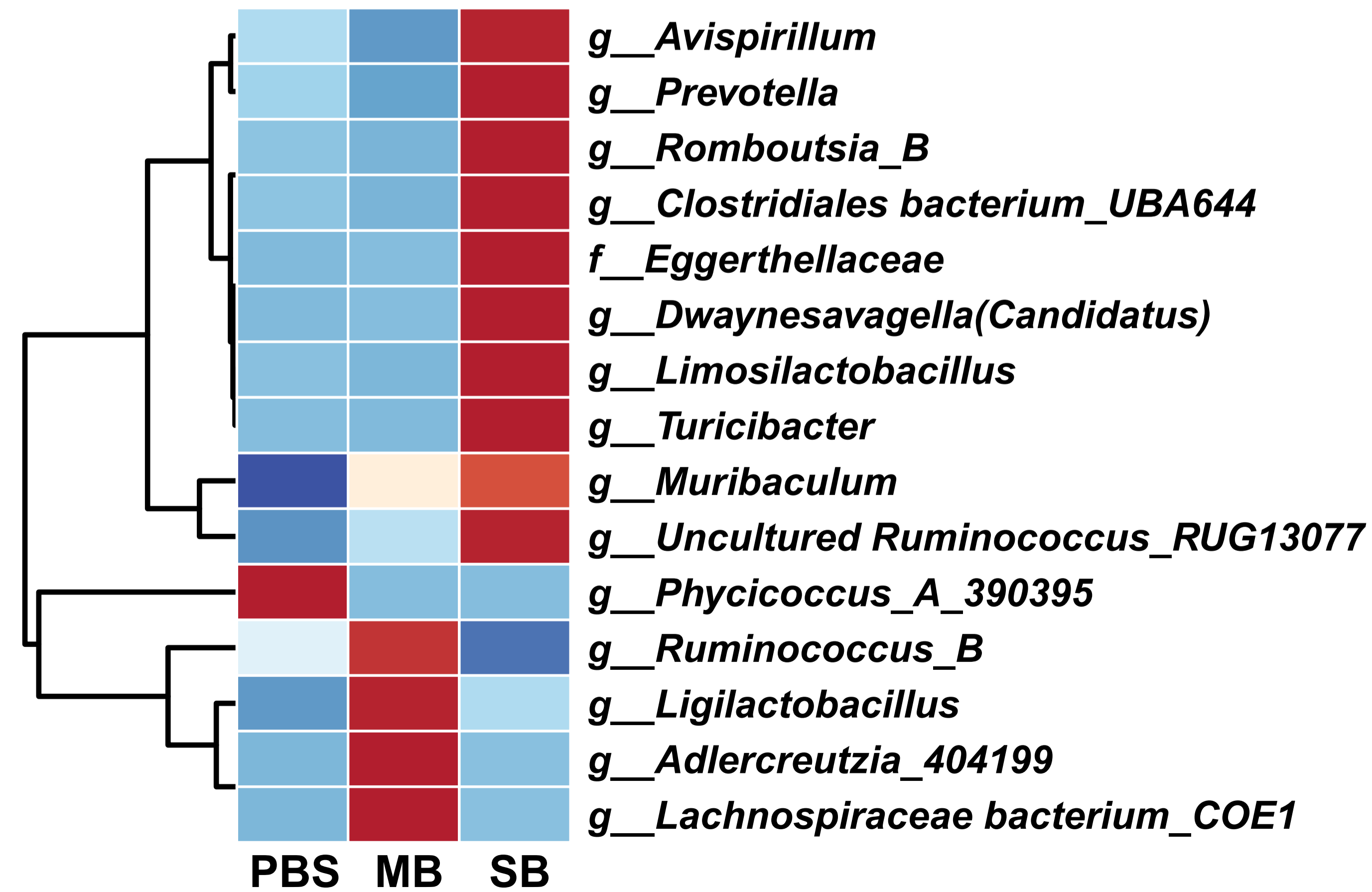

C

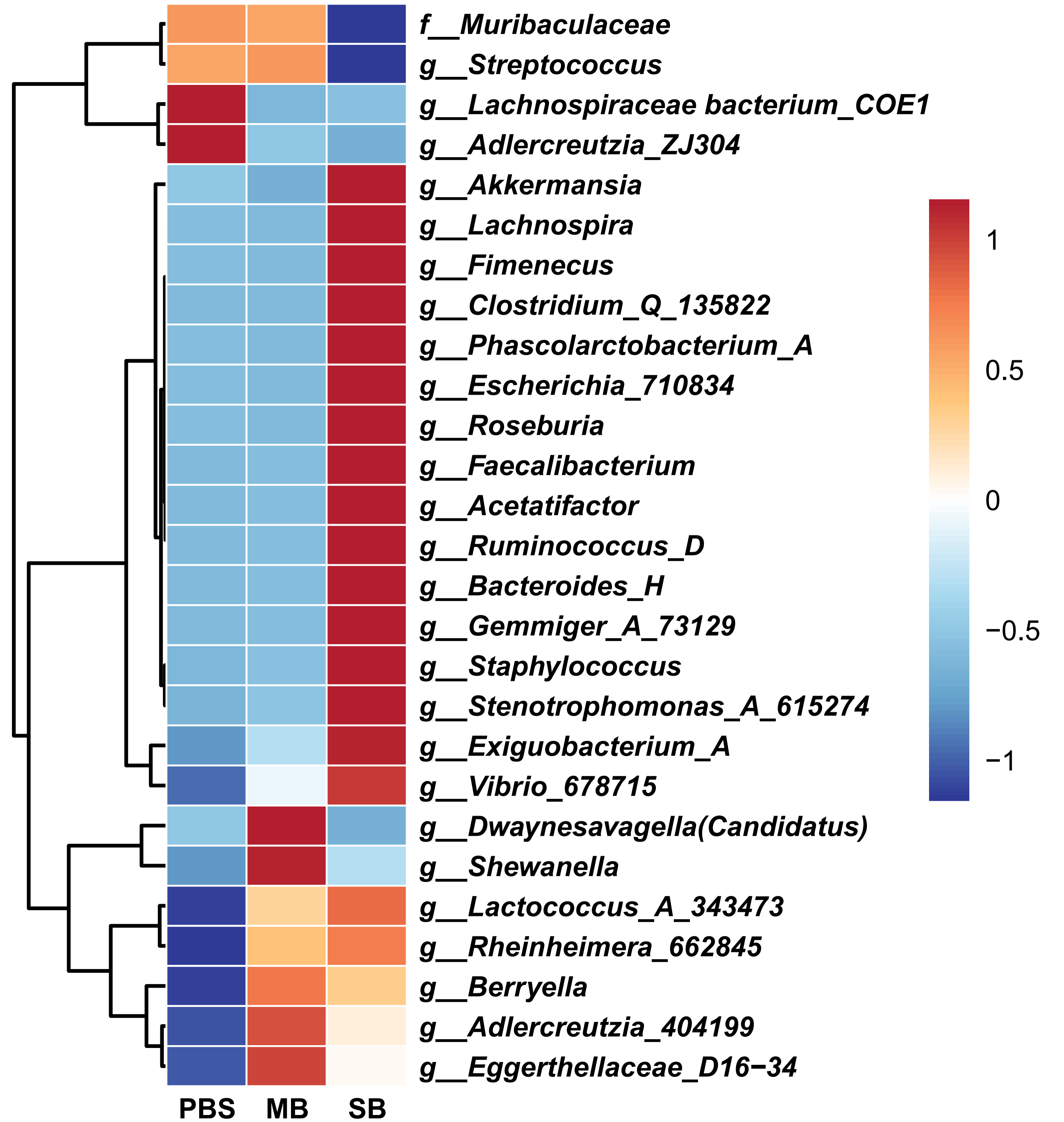

Supplement: Supplementary file 1 [file nutrients-16-02052-s001.zip › figure S6.pdf]
